# Seizures in an immunocompromised adolescent: a case report

**DOI:** 10.1186/s13256-015-0660-2

**Published:** 2015-08-28

**Authors:** Vipula R Bataduwaarachchi, Nirmali Tissera

**Affiliations:** 1 Department of Pharmacology and Pharmacy, Faculty of Medicine University of Colombo, PO Box 271, Kynsey Road, Colombo 8, Sri Lanka; 2 Department of Medicine, National Hospital, Ward Place, Colombo, Sri Lanka

## Abstract

**Introduction:**

Tuberculosis is a progressive and disabling infection predominantly seen in low-income and middle-income countries. Immunocompromised patients are at a higher risk of contracting tuberculosis than the healthy population. The presentation may also be atypical, leading to delay in diagnosis. We report the first case of tuberculous cerebral vasculitis presenting with epilepsia partialis continua.

**Case presentation:**

A 17-year-old adolescent boy of Sri Lankan Moor heritage was taking long-term immunosuppressants for nephrotic syndrome. He presented to hospital with focal fits affecting his left arm. He later developed choreiform movements of the same arm, progressing to epilepsia partialis continua and weakness. The gradually evolving focal neurological signs and underlying immunosuppression raised the possibility of localized cerebral infection or inflammation. Analysis of his cerebrospinal fluid showed lymphocytosis with normal cellular morphology. Magnetic resonance imaging was suggestive of progressive vasculitic infarctions of the cerebral cortex and basal ganglia. There was no evidence of active autoimmune or viral disease on hematological investigations, but molecular amplification detected *Mycobacterium tuberculosis* in his cerebrospinal fluid. Although our patient had been established on isoniazid preventive treatment for eight months before the episode, tuberculosis was nonetheless considered to be the most likely cause of the cerebral vasculitis. He was treated with a trial of anti-tuberculosis treatment, including streptomycin and adjunctive steroids, and made an uneventful recovery.

**Conclusion:**

Clinicians should have a high index of suspicion for tuberculosis infection in patients with compromised immunity and other risk factors. The pathophysiological mechanisms underpinning cerebral vasculitis and epilepsia partialis continua are not completely understood. The efficacy of isoniazid prophylaxis in patients with immune suppression warrants further study. We present a regimen that successfully treated tuberculous cerebral vasculitis.

## Introduction

Tuberculosis (TB) is a progressive and disabling infection caused by *Mycobacterium tuberculosis* that is predominantly seen in low-income and middle-income countries. It primarily affects the lungs, but it can affect any other organ system, including the central nervous system (CNS). Tuberculous vasculitis is a chronic immune-mediated vasculitis induced by *M. tuberculosis* that can affect blood vessels of all sizes.

Retinal vasculitis commonly affects the retinal veins and may cause retinal hemorrhage and neovascularization that is sufficient to severely impair vision [1]. Tuberculous skin manifestations due to vasculitis predominantly present as erythema nodosum and nodular vasculitis [2]. Tuberculous aortitis is rare, but can lead to aneurysm formation and rupture [3, 4]. Coronary arteritis due to tuberculous vasculitis is extremely rare but can cause serious complications, such as myocardial infarction, arrhythmia, and sudden death [5]. A rare case of tuberculous vasculitis and nasal septal perforation has been reported in the literature, illuminating a diagnostic dilemma caused by TB [6], in that both TB and granulomatosis with polyangiitis (Wegener’s granulomatosis) can destroy the nasal septum, giving rise to a similar clinical picture. Tuberculous enteritis was found in 87.5 % of cases diagnosed with abdominal TB in a study conducted among Indian patients [7].

Cerebral TB has a spectrum of presentations, such as meningitis, intracranial tuberculoma, and spinal tuberculous arachnoiditis, that is based on the tissues affected and the underlying pathophysiological process [8]. A case of tuberculous meningovasculitis resulting in hyperintense left-sided subcortical lesions on magnetic resonance imaging (MRI) consistent with infarction has been reported [9]. Cerebral venous sinus thrombosis is reportedly another rare complication of TB meningitis [10].

Immunocompromised patients are at a higher risk of TB infection than the healthy population. The presentation of TB infection in an immunocompromised individual may also be atypical, potentially delaying diagnosis [11]. The diagnosis of tuberculous cerebral vasculitis cannot be made by physical examination and imaging; laboratory and microbiological confirmation is also essential. Nonetheless, a high index of clinical suspicion is vital to guide the physician to select the correct investigations. Because of the complexity of its presentation and the difficulties that might arise as a result of delayed diagnosis, it is important to have a full understanding of the different presentations of TB in high-risk populations. Here, we report what we believe to be the first case of tuberculous cerebral vasculitis presenting as epilepsia partialis continua in a young man with nephrotic syndrome.

## Case presentation

A 17-year-old adolescent boy of Sri Lankan Moor heritage who was taking long-term potent immunosuppressants was admitted to the Emergency Medical Unit of our institution with focal fits affecting his left arm, accompanied by secondary generalization leading to status epilepticus. The postictal period was complicated by periodic drowsiness. He developed choreiform movements of the same arm on the second day of admission, and the convulsions persisted despite treatment with anticonvulsants. On the fifth day, he developed weakness of his left arm without sensory impairment. On the tenth day, he developed continuous abnormal seizure-like movements of his left arm lasting for hours, suggestive of epilepsia partialis continua. Our patient remained afebrile throughout the illness, and he denied any headache or photophobia. He was taking prednisolone 10mg and mycophenolate mofetil 500mg once daily, and tacrolimus 5mg twice daily, as maintenance treatment for steroid-resistant nephrotic syndrome. Our patient had been taking 300mg isoniazid (INAH) preventive therapy (IPT) for 8 months as a result of his potent immunosuppressant regime and the high prevalence of TB among the urban Moor population, which his nephrologist judged placed him at high risk of TB infection.

Our patient’s hemodynamic parameters and random blood sugar and electrolyte concentrations were within normal limits. Results of a general examination were normal except a Cushingoid appearance suggestive of long-term corticosteroid use. He had no neck stiffness, rash, or neurocutaneous manifestations. Examination of his optic fundi was normal. On the first day of admission he developed slurred speech. His arm weakness was suggestive of an upper motor neuron-type paresis. Taken together, his symptoms and signs were suggestive of a subacute progressive pathology localized to the motor cortex representing his left upper limb. The differential diagnoses at this point were meningoencephalitis with abscess formation, cerebral lupus, or cerebral vascular occlusion.

Tacrolimus was stopped immediately on admission because of the possibility of tacrolimus-induced CNS toxicity. A complete blood count and liver function test results were normal. His level of inflammatory markers, erythrocyte sedimentation rate, and C-reactive protein concentration were also normal. Serum anti-nuclear antibody and double-stranded DNA tests were negative, making cerebral lupus less likely. His previous renal histology showed diffuse mesenchymal proliferation with segmental sclerosis and patchy chronic tubulointerstitial changes in some glomeruli; however, his serum creatinine concentration and a urine analysis were normal. His serum calcium and magnesium concentrations were within their normal ranges. A cerebrospinal fluid (CSF) analysis showed normal protein and glucose concentrations, with a scanty lymphocytic infiltrate with normal cellular morphology. Electroencephalography confirmed focal seizure activity with secondary generalization. Blood, urine, and CSF cultures were negative for bacterial growth.

Chronic or atypical infection was suspected considering his immunosuppressed state. A tuberculin skin test was measured as 12 mm, which was considered significant in the context of his underlying immunosuppression. Acid-fast bacilli were not isolated in his sputum, urine, or CSF. Serum enzyme-linked immunosorbent assay (ELISA) screening for human immunodeficiency virus (HIV) and Venereal Disease Research Laboratory screening for syphilis were both negative, while an ELISA of his CSF was negative for Japanese encephalitis virus, herpes simplex virus 1 and 2, and cytomegalovirus immunoglobulin M.

**Fig. 1:**
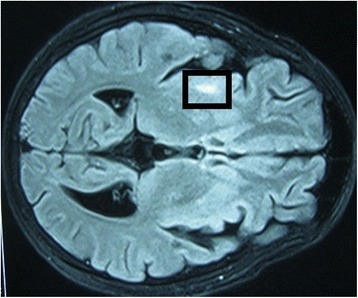
Magnetic resonance image: transverse section of the brain on the third day of admission. This section shows high T2 signals in the right hippocampal gyrus, right putamen, and the head of the caudate nucleus, suggestive of infarctions

**Fig. 2:**
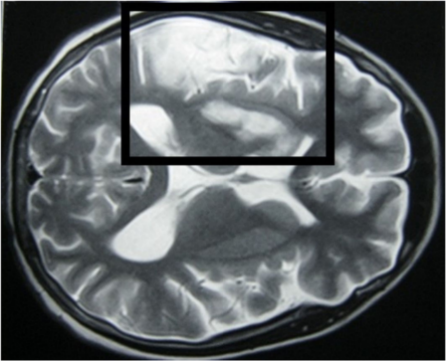
Magnetic resonance image: transverse section of the brain 1 month after the onset of illness. This section shows new involvement of the right cerebral cortex (right middle meningeal arterial territory) with vasogenic edema

**Fig. 3:**
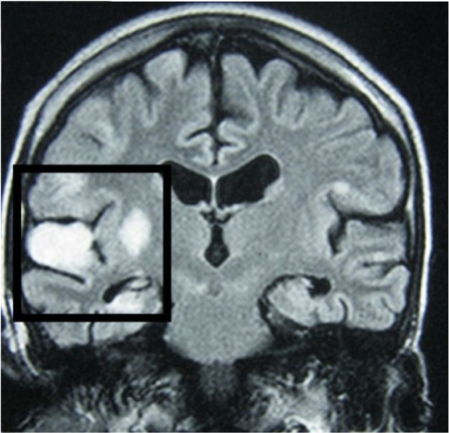
Magnetic resonance image: coronal section of the brain 1 month after the onset of illness. This section shows the involvement of the right cerebral cortex and hippocampus due to multiple cerebral and basal ganglia infarctions

**Fig. 4:**
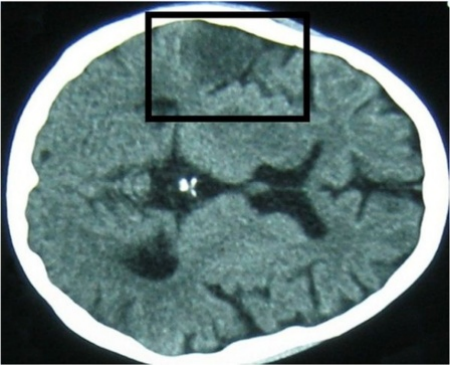
Computed tomography image: transverse section of the brain 1 month after the onset of illness. This section shows the infarctions of the right cerebral cortex

Non-enhanced computed tomography (CT) of his brain showed no abnormalities on the first day of admission. On the third day of admission, T2-weighted MRI of his brain revealed high intensity signals in his right hippocampal gyrus, right putamen, and the head of the caudate nucleus, suggestive of multiple infarctions (Fig. 1). Magnetic resonance angiography and venography were normal, however, excluding the possibility of macrovascular occlusion. MRI was repeated 1 month later because of the progressive nature of his symptoms and signs, and showed an increase in the size of original lesions and the development of new areas of involvement in the cerebral cortex with associated vasogenic edema (Figs. 2 and 3). At the same time, a repeat CT scan showed infarctions in the cortex but no basal ganglia involvement (Fig. 4). Even though areas of infarction caused by a single event may have evolving radiological features, the progressive clinical manifestations we observed favored an on-going, persistent, and escalating brain injury. We repeated the lumbar puncture, and CSF samples were sent for TB and fungal studies. Polymerase chain reaction (PCR) testing for TB was positive in two independent samples performed at two different laboratories. A quantitative PCR assay is reportedly an accurate and reliable means of quantitative detection of *M. tuberculosis* DNA in CSF samples owing to the development of a new-mutation plasmid as an internal control [12]. Another study reported that the technique was less sensitive but more specific for the diagnosis of tuberculous meningitis in individuals with HIV infection, individuals living in a TB-endemic setting, particularly when a centrifuged CSF pellet is used (sensitivity 62 %, specificity 95 %) [13]. TB was considered the most likely cause for cerebral vasculitis in our patient based on these findings. This case highlights the effectiveness of TB PCR in the diagnosis of CNS TB, obviating the need for brain biopsy.

In this case, MRI was supportive of our diagnosis and was superior to CT for detecting CNS TB, which concurs with the findings of previous studies [14, 15]. A hypercoagulable state is reported to increase the risk of thrombosis in childhood TB meningitis [16], but the effectiveness of anticoagulants for the prevention of cerebral infarctions caused by infective vasculitis has not been properly evaluated. The diagnosis of TB in a patient with underlying immunosuppression despite latent anti-TB treatment (LTBT) raises the possibility that IPT was ineffective in this clinical context. In a previous study of IPT, only one patient from the group treated with INAH was infected with TB during the study period, compared with ten in the non-INAH group [17]. Nonetheless, resistance to INAH, and rifampicin when it is used as an alternative, has been reported [18, 19]. Evidence for the benefit of IPT as LTBT is lacking, and a multicenter trial of IPT in an endemic area is warranted [20]. Because TB is endemic in Sri Lanka, particularly in urban areas, it is plausible that the strain of *M. tuberculosis* causing infection in this case was resistant to INAH.

A variety of approaches to the treatment of CNS TB have been taken, including combination therapy of an anti-TB treatment (ATT) with an immunosuppressant such as oral prednisolone, parenteral methylprednisolone pulse therapy, or cyclophosphamide monotherapy; however, their efficacy has not been properly established because of the lack of an adequate cohort [11, 21]. In this case, we elected to commence a 12-month course of ATT as per the World Health Organization guidelines. This consisted of a 2-month intensive phase of treatment with oral INAH 5 mg/kg/day, rifampicin 10mg/kg/day, and pyrazinamide 30mg/kg/day, and intramuscular streptomycin 20mg/kg/day, followed by a 10-month maintenance phase of INAH and rifampicin. Adjunctive methylprednisolone pulses (1g/day for 3 days) were also given, followed by an oral maintenance dose (1mg/kg once in the morning) for 1 month and then tapered off over another month. His anticonvulsant therapy was sodium valproate 200mg three times daily and clobazam 10mg twice daily. His nephrologist restarted immunosuppression for the underlying nephrotic syndrome; there was no recurrence of renal disease on azathioprine 50mg once daily.

The combination of treatments put our patient at increased risk of liver injury. We would have stopped therapy if there had been any evidence of hepatotoxicity, and would have started him on bridging therapy with non-hepatotoxic ATT until the liver injury had resolved. We also monitored his renal function to guide any dose adjustments. Regular monitoring found that his serum creatinine concentration and liver function test results remained normal throughout his treatment regime. He responded well to treatment—the convulsions resolved rapidly once ATT had started. Surveillance imaging showed no further progression of the cerebral lesions, further supporting the diagnosis and choice of treatment strategy. The weakness in his left arm persisted, however, suggesting that there had nonetheless been a permanent brain injury. He was subsequently able to achieve a partial improvement in left arm power after intensive rehabilitation.

## Conclusion

Our patient, who was taking immunosuppressants for nephrotic syndrome, developed cerebral TB vasculitis causing multiple cortical and basal ganglia infarctions despite LTBT. The effectiveness of LTBT in immunocompromised patients requires further evaluation in large-scale studies. We propose that our patient was infected with an INAH-resistant *M. tuberculosis* species. An ATT regime combined with steroids adequately controlled the progression of CNS TB vasculitis in this case. Further studies are needed to understand the pathophysiologic basis for the association between cerebral vasculitis and epilepsia partialis continua.

## Consent

Written informed consent was obtained from the patient and his legal guardian(s) for publication of this case report and any accompanying images. A copy of the written consent is available for review by the Editor-in-Chief of this journal.

## Abbreviations

ATT: anti-tuberculosis therapy
CNS: central nervous system
CSF: cerebrospinal fluid
CT: computed tomography
ELISA: enzyme-linked immunosorbent assay
HIV: human immune deficiency virus
INAH: isoniazid
IPT: isoniazid preventive therapy
LTBT: latent anti-tuberculosis treatment
MRI: magnetic resonance imaging
TB: tuberculosis
